# Autoimmune Pancreatitis: From Pathogenesis to Treatment

**DOI:** 10.3390/ijms232012667

**Published:** 2022-10-21

**Authors:** Enrico Celestino Nista, Sara Sofia De Lucia, Vittoria Manilla, Tommaso Schepis, Antonio Pellegrino, Veronica Ojetti, Giulia Pignataro, Lorenzo Zileri dal Verme, Francesco Franceschi, Antonio Gasbarrini, Marcello Candelli

**Affiliations:** 1 Department of Medical and Surgical Sciences, Università Cattolica Sacro Cuore, Fondazione Policlinico Universitario A. Gemelli IRCCS, 00168 Rome, Italy; 2 Department of Emergency, Anesthesiological, and Reanimation Sciences, Università Cattolica Sacro Cuore, Fondazione Policlinico Universitario A. Gemelli IRCCS, 00168 Rome, Italy

**Keywords:** autoimmune pancreatitis, AIP-1, AIP-2, IgG4-related disease

## Abstract

Autoimmune pancreatitis (AIP) is a rare disease. The diagnosis of AIP is difficult and should be made by a comprehensive evaluation of clinical, radiological, serological, and pathological findings. Two different types of AIP have been identified: autoimmune pancreatitis type 1 (AIP-1), which is considered a pancreatic manifestation of multiorgan disease related to IgG4, and autoimmune pancreatitis type 2 (AIP-2), which is considered a pancreas-specific disease not related to IgG4. Although the pathophysiological conditions seem to differ between type 1 and type 2 pancreatitis, both respond well to steroid medications. In this review, we focused on the pathogenesis of the disease to develop a tool that could facilitate diagnosis and lead to the discovery of new therapeutic strategies to combat autoimmune pancreatitis and its relapses. The standard therapy for AIP is oral administration of corticosteroids. Rituximab (RTX) has also been proposed for induction of remission and maintenance therapy in relapsing AIP-1. In selected patients, immunomodulators such as azathioprine are used to maintain remission. The strength of this review, compared with previous studies, is that it focuses on the clear difference between the two types of autoimmune pancreatitis with a clearly delineated and separate pathogenesis. In addition, the review also considers various therapeutic options, including biologic drugs, such as anti-tumor necrosis factor (TNF) therapy, a well-tolerated and effective second-line therapy for AIP type 2 relapses or steroid dependence. Other biologic therapies are also being explored that could provide a useful therapeutic alternative to corticosteroids and immunosuppressants, which are poorly tolerated due to significant side effects.

## 1. Materials and Methods

Articles were found in the PubMed, Scopus, and EMBASE databases using the following search terms: “autoimmune pancreatitis,” “International Consensus Diagnostic Criteria,” “epidemiology,” “pathogenesis,” “glucocorticoids,” “azathioprine,” “rituximab,” “anti-tumor necrosis factor,” “IgG4-associated disease.” Authors reviewed English language articles individually for relevance and results were compared to include only the most relevant articles. Letters, comments, and opinions were not considered in the search.

## 2. Introduction

### 2.1. Definitions

Autoimmune pancreatitis (AIP) is defined by the International Consensus Diagnostic Criteria 2010 (ICDC) as a specific form of pancreatitis characterized by obstructive jaundice with or without pancreatic masses, lymphoplasmacytic infiltrate and fibrosis, and a marked response to steroids [1]. AIP can be divided into type 1 (AIP-1), also called lymphoplasmacytic sclerosing pancreatitis (LPSP), and type 2 (AIP-2), also called idiopathic ductal centric pancreatitis (IDCP) [2]. AIP-1 and AIP-2 differ in terms of epidemiology, pathogenesis, histologic pattern, and natural history.

**Figure 1:**
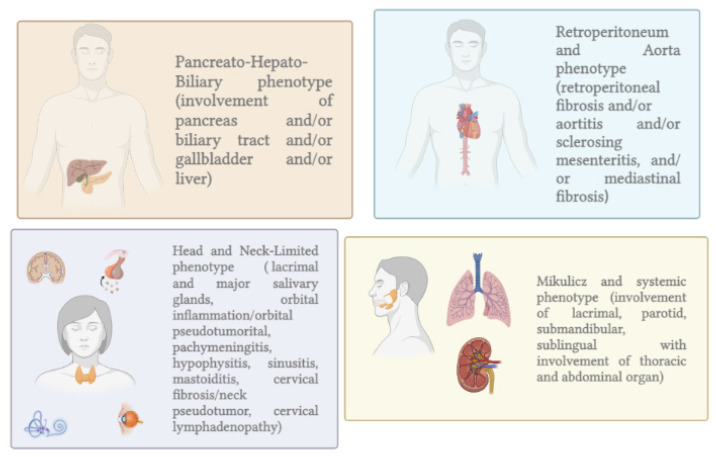
IgG4-RD phenotypes [10].

AIP-1 is the pancreatic manifestation of IgG4-related disease (IgG4-RD) characterized by lymphoplasmacytic infiltration with more than ten IgG4-positive plasma cells per high-power field (HPF), storiform fibrosis, and obliterative phlebitis [3]. Clinically, IgG4-RD is a systemic disease that can affect virtually all organs. AIP-1, retroperitoneal fibrosis, chronic periaortitis, autoimmune hypophysitis, sclerosing cholangitis, Riedel’s thyroiditis, and Mikulicz disease are the most common manifestations of IgG4-RD (Figure 1) [4].

Based on the distribution of organ involvement, four characteristic IgG4-RD phenotypes can be distinguished: pancreatic-hepatobiliary disease; retroperitoneal fibrosis and/or aortitis; disease confined to the head and neck; Mikulicz syndrome with systemic involvement. According to the study by Wallace et al., pancreatic–hepatobiliary disease is the most common phenotype. Female and Asian patients are most common in the hand–neck group. Patients with Mikulicz syndrome have the highest serum IgG4 levels, whereas patients with retroperitoneal fibrosis and/or aortitis have the lowest serum IgG4 levels [5]. In contrast, AIP-2 is not a systemic disease, and the pancreas is the only organ affected. The fibro-inflammatory process mainly involves the pancreatic ducts with neutrophilic infiltration of the medium and small ducts. The presence of granulocytic epithelial lesions (GELs) in the medium and small ducts and in the acini is the pathognomonic sign [6,7]. In 15–30% of cases, AIP-2 is associated with inflammatory bowel disease (IBD), typically ulcerative colitis. Clinically, AIP-2 is more common in young patients, and the typical clinical presentation is acute pancreatitis. Recently, a third type of AIP has been described and designated as not otherwise specified (NOS) [8]. This is not a true pathologic entity but a form of AIP that cannot be classified as type 1 or type 2. It could be IgG4-seronegative AIP-1, undiagnosed AIP-2, or an overlap syndrome. According to Ikeura et al., AIP NOS accounts for 16% of AIP diagnosis [9].

### 2.2. Epidemiology

The overall prevalence and incidence of AIPs are unknown. Thanks to the widespread recognition of AIP and IgG4-RD and the development of diagnostic criteria, the number of total and newly diagnosed AIP patients in Japan has increased rapidly, with the fourth nationwide epidemiological survey conducted in 2016. Compared with 2011, the number of newly diagnosed patients has more than doubled [11]. According to this study, the prevalence of AIP increased significantly compared with a previous study (4.6 vs. 10.1 per 100,000 patients) [12]. The annual incidence was 3.1 per 100,000 people. The sex ratio of males to females was 2.94. The mean age at diagnosis was 64.8 years.

A retrospective review by the AIP, examining 15 different institutions from 8 countries, found that the LPSP variant was detected much more frequently than the IDCP variant. The mean age of all patients with LPSP was higher than that of IDCP patients (61.6 and 44.8 years, respectively), and the two groups did not differ in terms of gender [13]. An international multicenter survey of AIP involving ten different countries found regional and ethnic differences between the two types of AIP. Accordingly, the proportion of patients diagnosed with AIP-2 was lower in Asia than in Europe and North America [14].

In Italy, the prevalence of AIP types is similar to the rest of the world: 61% of patients were classified as AIP-1, 28% as AIP-2, and 11% as AIP-NOS. The proportion of men was 66.9% in patients with AIP-1 and 54.2% in AIP-2. The median age at diagnosis was significantly lower in AIP-2 (48 versus 62.5 years) [15].

### 2.3. Diagnosis

The first diagnostic criteria for AIP were proposed by the “Japan Pancreas Society” in 2002 and revised in 2006. The criteria considering radiological, serological, and histopathological elements are described in Table 1 [16]. However, the Japanese criteria for AIP focused primarily on the radiologic features of autoimmune pancreatitis and ignored two other fundamental aspects of this pathology: a clear response to corticosteroid therapy and involvement of other organs. The Mayo Clinic proposed new criteria in which involvement of other organs and response to therapy were the newly introduced parameters. Five main features of AIP were identified: histology, imaging, serology, involvement of other organs, and response to steroid therapy, referred to as HISORt [17,18].

In 2011, the International Consensus Diagnostic Criteria (ICDC) for AIP were developed after a review of existing criteria, including JPS (2002, 2006), HISORt (2006, 2009), Korean (2007), Asian (2008), Mannheim (2009), and Italian (2003, 2009) [1]. The ICDC distinguished between AIP-1 and AIP-2 and divided each criterion into levels of evidence for both: typical/highly suggestive for AIP and indeterminate/suggestive for AIP, as shown in Table 2 and Table 3 [1].

The ICDC is currently the standard for the diagnosis of AIP and is more effective in diagnosing this disease than previously published systems [19].

Ultimately, the Unifying Autoimmune Pancreatitis Criteria (U-AIP) provided an additional and accurate diagnostic approach for AIP [20]. Although these criteria have the limitation of not being able to distinguish between type 1 and type 2 of AIP, the U-AIP criteria have the advantage of being able to distinguish cases with equivocal diagnoses in rare forms; the criteria are summarized in Table 4.

A major difference in diagnosis between the two types of pancreatitis is that in AIP-1, histologic examination is not essential to make the diagnosis. In contrast, in AIP-2, histologic examination is usually required to demonstrate neutrophil infiltration into the ducts or acini [21].

Chiari et al. proposed a revision of the HISORt diagnostic criteria to develop a clinical strategy to distinguish AIP from pancreatic adenocarcinoma (PdaC). Patients were divided into groups in whom AIP was highly probable, those in whom PdaC was highly probable, and those in whom the diagnosis was uncertain [22].

#### Laboratory Findings in Aip

According to evidence-based recommendations published in 2020 by United European Gastroenterology (UEG) and the Swedish Society of Gastroenterology (SGF), serum IgG4 level alone is not sensitive and specific enough to establish the diagnosis of IgG4-related gastrointestinal disease but should be measured if the disease is suspected (grade 2C; strong agreement) [23]. However, not all individuals affected by AIP 1 have elevated serum IgG4 levels; according to the meta-analysis performed by Lian et al., both serum IgG4 and IgG had high specificity and relatively low sensitivity for the diagnosis of AIP, so neither is the ideal biomarker [24]. The cut-off value for IgG4 ranged from 130 mg/dL to 140 mg/dL. Other common AIP-1 laboratory findings include:

- Electrophoresis changes: Jacobs et al. suggested that a characteristic focal band bridging the β- and γ-fractions in serum protein electrophoresis may be an initial serologic clue to IgG4–RD research [25].
- Hypocomplementemia was found in 36% with AIP by Muraki et al. [26].
- ANA and RF positivity [27].
- Other autoantibodies: efforts have focused on finding organ-specific autoantibodies because they may have pathogenetic significance in triggering the disease, but the truth is that most of them are not very common in AIP or are seen only in low titers. The diverse antibodies include anti-lactoferrin (Anti-LF), anti-carbonic anhydrase II (Anti-CA-IIAb), anti-carbonic anhydrase IV (Anti-CA-IVAb), anti-pancreas secretory trypsin inhibitor (anti-PSTI), anti-trypsinogen, anti-amylase alpha, anti-heat shock protein 10 (anti-HSP10), and anti-plasminogen-binding protein peptide (anti-PBP); none of these autoantibodies can be considered disease-specific [27].

In recent years, other serological markers have been found that could help in AIP-1 diagnosis. Some of them could also be helpful in monitoring disease activity and response to treatment and are included again in the list below:

1. A quantitative polymerase chain reaction (qPCR) for the analysis of IgG4+ RNA molecules was developed by Tabibian et al. in 2016 and used in a prospective case-control study of patients at two European medical centers. It was found that IgG4+ RNA could be a more accurate diagnostic marker than IgG4 protein. However, further studies are needed to validate this method [28].
2. Circulating plasmablasts and CC-chemokine ligand 18 (CCL18) measured by flow cytometry and enzyme-linked immunosorbent assay (ELISA) are also cited as valuable biomarkers, not only for diagnosis, but also for disease monitoring, as indicated in the evidence-based UEG and SGF recommendations [23].
3. IFN-alpha and IL-33, which are involved in the development of IgG4-RD, have diagnostic value as markers of type 1 AIP and/or IgG4-RD, comparable to that of serum IgG4 concentration, as calculated by Minaga et al.; they also showed markedly decreasing levels after induction of remission by prednisolone [29].
4. Eosinophils and IgE levels are increased in a consistent number of AIP-1 patients [30,31].

So far, we have not found a valid biomarker for AIP-2, so biopsy is the only way to make the diagnosis and distinguish the focal form of PDAC.

## 3. Pathogenesis

### 3.1. Pathogenesis of AIP-1

**Figure 2:**
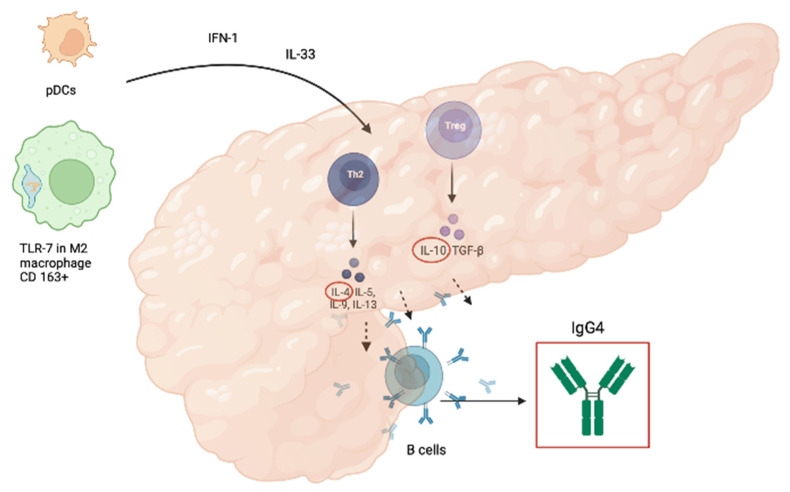
Pathogenesis of autoimmune pancreatitis type 1.pDCs: plasmacitoid dendritic cells, INF-1: interferon 1; Il-33, 4, 5, 9, 13, 10: intereleukin-33, 4, 5, 9, 13, 10; Th2: T helper 2 cells; Treg: regulatory T cells; TLR-7: toll-like receptor 7; CD 163+: cluster of differentiation 163+; TGF-β: transforming growth factor-β; IgG4: immunoglobuline G4.

The pathogenesis of AIP-1 is not yet fully understood, and many details are still unclear. The innate and adaptive immune systems are involved in the development of IgG4-RD (Figure 2).

Toll-like receptors are pattern recognition receptors (PRRs) that are activated by antigens such as intestinal bacteria and are an essential component of innate immunity [32]. Overexpression of certain types of Toll-like receptors (TLRs) in the pancreas and salivary glands of patients with AIP and IgG4-RD, underscores the central role of the innate immune system in the development of AIP-1 [33]. Ishiguro et al. documented the upregulation of TLR-7 in IgG4-RD when comparing salivary glands from AIP patients and healthy controls. TLR-7 expression was evident in CD 163+ M2 macrophages, which produce a large amount of interleukin-33 (IL-33) [34]. IL-33 is a nuclear cytokine of the IL-1 family that is constitutively expressed in tissues (epithelial cells of tissues exposed to the environment, fibroblastic reticular cells of lymphoid organs, and endothelial cells of human vessels) under basal conditions [35] and overexpressed in the presence of inflammation (e.g., exposure to viral pathogens, parasites, and environmental allergens), with activation of fibroblasts and consequent development of fibrosis [36,37].

The plasmacytoid dendritic cells (pDCs) may also play a key role in the pathogenesis of murine AIP and also human AIP/IgG4-RD. pDCs represent a small percentage of human immune cells but are the major cellular source of type 1 interferons (IFN-I) and play a key role in host defense against microbial infections [38,39]. Nevertheless, unregulated production of IFN-I by pDCs may underlie not only autoimmune pancreatitis but also other autoimmune diseases such as psoriasis and systemic lupus erythematosus (SLE) [40]. High circulating IFN levels are associated with a more severe course of these diseases [41].

In a mouse model of AIP, pDCs were shown to produce IL-33 by stimulation with type I interferons (IFN-I). Blocking IL-33-mediated signaling by a neutralizing antibody (Ab) to its receptor interleukin 1 receptor-like (also called ST2) was associated with a reduction in pancreatic inflammation and fibrosis development [42]. The pathogenic role of IL-33 is reflected in its ability to stimulate an aberrant Th2 response with subsequent production of fibrogenic cytokines, such as interleukin 4 (IL-4) and interleukin 13 (IL-13); concentrations of type 1 IFN and IL-33, appear to be increased in both tissue and serum from patients with IgG4-RD, suggesting the use of IL-33 as a novel IgG4-RD biomarker [29]. In a recent review by Minaga et al., it was analyzed that pDCs could be a potential therapeutic target.

Depletion of pDCs or neutralization of signaling pathways mediated by IFN-I and IL-33 prevented the development of experimental AIP in mice [43].

IL-33 is directly related to the mechanisms of adaptive immunity involved in the pathogenesis of AIP 1, as it is able to promote the activation of Th2 cells, type 1 helper T cells (Th1), innate group 2 lymphoid cells (ILC2s), but also regulatory T cells (Tregs) [44]. Grados et al. found increased levels of circulating regulatory T, TH2, TH17, and CD4+ T cells, CXC chemokine receptor 5 (CXCR5+), and programmed death-1 (PD1+) T follicular helper cells (Tfh) in a multicenter study of 28 patients with IgG4-RD compared with healthy controls and with patients with primary Sjogren’s syndrome [45]. Similar results were obtained by Yoh Zen et al., who documented a Th2-predominant response and the presence of a significant number of Tregs in AIP-affected tissues, which is unusual in autoimmune diseases but common in allergic reactions [46,47]. In IgG4-RD, the switch of immunoglobulins to the IgG4 subclass is controlled by IL-4 and Il-10, which are produced by Th2 and Tregs, respectively. This mechanism is activated upon prolonged exposure to an antigen and reduces inflammatory phenomena [48,49]. Clinically, this explains fibrosis development as a result of fibroblasts stimulated by IL-4 and perhaps justifies the large prevalence of IgG4-RD in allergic patients, but this correlation should be confirmed [50,51]. The role of IgG4 in the development of AIP-1 and IgG4-RD is still unclear. IgG4 is considered to be an anti-inflammatory Ab because of its poor ability to form large immune complexes and activate complement [52]. Low C3 and C4 levels are a common laboratory finding in AIP-1 and recent studies have demonstrated the presence of immune complexes in affected tissues. On the one hand, patients with AIP-1 and high immune complexes have increased IgG1 levels and decreased C3 and C4 levels, suggesting the prominent role of IgG1 in activation of the complement system; on the other hand, immune complexes precipitated with polyethylene glycol in patients with IgG4-RD and hypocomplementemia showed the ability to activate complement through both the classical and mannose-binding lectin pathways. However, this does not preclude the role of IgG4 in complement system activation [53,54].

Assuming that the increased number of IgG4 is secondary to prolonged exposure to an antigen, as is the case with allergic reactions, the antigens involved in the development of AIP-1 have not yet been defined. In the era of the gut microbiome, the interactions between the microbiota and our immune system have been extensively studied [55].

Jian Ji et al. showed that butyrate from commensal microbiota stimulates the polarization of M2 macrophages [56]; Kamata et al. suggested a role of gut dysbiosis in the activation of plasmacytoid dendritic cells (pDCs), leading to increased sensitivity to experimental AIP [57]. In addition, Kamata et al. analyzed the intestinal microbiota of three patients with AIP before and after steroid treatment and showed complete disappearance of Klebsiella pneumoniae in two cases after prednisolone administration [58]. Yamaki et al. produced distinct lesions in the exocrine pancreas of mice immunized with a mixture of an extract of pooled pancreas from syngeneic mice and the capsular polysaccharide of Klebsiella pneumoniae type 1 Kasuya strain (CPS-K), resulting in histologic changes, characterized by infiltration with lymphocytes, plasma cells, and other mononuclear cells, degeneration and lysis of acinar cells, destruction of lobular architecture, and replacement by adipose tissue and fibrous connective tissue, preserving endocrine islets [59]. Kamata et al. repeated a similar experiment with a well-established mouse model of AIP type 1, assessing a pathologic score and confirming that oral administration of heat-killed K. pneumoniae promoted the development of pancreatitis [58]. However, the autoimmunity theory is not abandoned because it is known that carbonic anhydrases (Cas), lactoferrin (LF), a red iron-binding protein, are mainly present in the secretion of body fluids such as pancreatic juice and that anti-LF and anti-CA II, anti-CA I, and anti-CA II, and anti-CA IV are common nonspecific autoantibodies found in patients with AIP, but the role of these autoantibodies in the pathogenesis of AIP is still unclear. Recently, Song-Chou Hsieh et al. described a significant number of autoantibodies associated with IgG4-RD [60]. Among these autoantibodies, antibodies against plasminogen-binding protein (PBP) of Helicobacter pylori play an important role.

This antibody was found in a large number of patients with AIP. Frulloni et al. found anti-PBP in 93% of AIP patients [61], and, probably for this reason alone, the role of H. pylori infection in the pathogenesis of autoimmune pancreatitis is largely controversial. However, the association between H. pylori infection and autoimmune pancreatitis was recently disputed by Youssefi et al. [62].

### 3.2. Pathogenesis of AIP-2

The pathogenesis of AIP-2 is less known compared to AIP-1 [21]. AIP-2 is characterized by neutrophil-rich inflammation. In particular, granulocytic epithelial lesions (GELs), consisting of neutrophilic ductal infiltration are the major histologic finding of the disease [63]. A possible role of Th-17 in the development of the disease was suggested [64]. Th-17 is a novel subset of CD4+ effector T cells recently identified and characterized by expression of the transcription factor retinoic acid receptor-related orphan receptor C (RORC) and secretion of key cytokines, including IL-17A, IL-21, IL-22, and IL-23. However, the mechanism leading to the infiltration of Th17 cells in the periductal pancreatic tissue of AIP-2 patients remains to be elucidated. A genetic hypothesis is proposed by Dong et al., who found the presence of mutations of the multiple endocrine neoplasia 1 (MEN1) and polycystic kidney and liver disease 1 (PKHD1) gene in AIP-2 [65]. Recently, overexpression of IL-8 was detected in the affected pancreatic tissues of patients with AIP-2. The main functions of IL-8 are angiogenesis and chemotaxis of neutrophils, allowing them to migrate to the site of inflammation [66]. This overexpression has also been noted in ulcerative colitis and is often associated with AIP-2 [67,68]. A recent study also demonstrated overexpression of programmed death ligand 1 (PDL1) in AIP-2, suggesting that it is an immunologic biomarker, but its role in pathogenesis has not yet been investigated [69].

## 4. Therapy

Experts from different countries have proposed international recommendations for the treatment of AIP worldwide. Since 2016, according to these consensus statements, the autoimmune pancreatitis therapy is reserved for symptomatic patients in whom: (1) pancreatic involvement: e.g., obstructive jaundice, abdominal pain, back pain; (2) other organ involvement (OOI): e.g., jaundice due to biliary stricture. Alternatively, it may be suggested in asymptomatic patients if they have any of the following features: (1) persistent pancreatic mass on imaging; (2) persistent liver test abnormalities in a patient with associated IgG4-related sclerosing cholangitis (IgG4-SC) [70].

The UEG has given a similar indication but also recommends treatment of symptomatic patients for subclinical conditions that may lead to severe or irreversible organ failure [23]. According to current literature, 10–25% (up to a maximum of 55% from the study by Hart et al.) of patients with IgG4-RD show spontaneous resolution of symptoms without medical treatment [70]. According to the international consensus for the treatment of autoimmune pancreatitis mentioned above, steroids are the first-line agent to use in all patients with active AIP. In the case of contraindications to the use of steroids, rituximab is the only agent with recognized efficacy in inducing remission as a single agent, whereas other steroid-sparing therapies (e.g., thiopurines) are not very effective when used as monotherapy [70].

Clinical complete remission is defined as disappearance of symptoms, normalization of serum IgG or IgG4 levels, and disappearance of typical enlargement of the pancreas and irregular narrowing of the pancreatic duct. Incomplete remission means that only 1 or 2 of these categories are met. Symptomatic and radiographic remission can be observed within 2 to 4 weeks after initiation of steroid therapy [71]. Relapse of AIP is the recurrence of symptomatic, serological, radiological, or histological abnormalities after complete or incomplete remission of AIP.

Some patients with AIP-1 may require maintenance therapy with low-dose glucocorticoids or immunosuppressive agents or rituximab after induction of remission [70]. To decide whether maintenance therapy is needed, it is important to determine disease activity based on imaging findings, serum IgG4 levels, and the presence or absence of extrapancreatic lesions [72].

Maintenance therapy is recommended in patients with AIP-1 who have diffuse enlargement of the pancreas, delayed radiographic remission, or persistently high serum IgG4 (>2xUNL) after treatment, or more than 2 OOIs (>2) or association with proximal IgG4-SC before treatment [70]. Suggested medications for maintenance therapy are low dose steroids (2.5–7.5 mg/day), immunomodulators, or rituximab. Although the duration of maintenance therapy is still controversial, Japanese experts recommend the use of low-dose glucocorticoid maintenance therapy for up to 3 years, and cessation of maintenance therapy should be planned in cases with radiological and serological improvement [73].

### 4.1. Steroids

Glucocorticoids (GC) have pleiotropic effects on the immune system. They can inhibit the initiation of the innate immune response, by inhibiting dendritic cells maturation and by dampening signal transduction downstream of pattern recognition receptors (PRRs); TLR signaling, for example, is subject to regulation by glucocorticoids at several points of signal transduction. Glucocorticoids also inhibit the expression of many pro-inflammatory cytokines many of which are involved in AIP pathogenesis. They can also regulate adaptive immunity by inhibiting lymphocyte activation and promoting lymphocyte apoptosis. At high concentrations, glucocorticoids also inhibit the production of B cells and T cells. Although the effect of glucocorticoid action is to reduce T cell activity, numerous studies suggest that glucocorticoids preferentially suppress the responses of TH1 cells and TH17 cells, while sparing or even promoting the function of TH2 cells and regulatory T (Treg) cells with subsequent production of the TH2 type cytokines IL4, IL10, and IL13. However, it does not seem to exacerbate diseases characterized by TH2 polarization, as already described for asthma and allergy [74].

According to UEG and SGF evidence-based recommendations, the initial posology of prednisone should be 0.6–0.8 mg/kg per day (typically 30–40 mg/die) for one month, evaluating response to treatment after 2–4 weeks. Afterward, prednisone dosage should be tapered by 5 mg every 2 weeks and suspended after 3–6 months [23]. In cases of refractory patients, a steroid mini-pulse treatment (2 courses of methyl-prednisolone 500 mg × 3 days with 4 days intervals) may be effective [70].

Although remission is reached easily in most cases of AIP after corticosteroid induction treatment, AIP relapse after or during initial treatment is common and represents a challenging problem in clinical practice. Patients treated successfully with steroid induction therapy can present a disease relapse in up to 33% of cases. In this setting, the maintenance of low-dose steroid therapy lasting longer than one year can reduce the risk of relapse. Patients with AIP-1 more commonly present relapses when compared with AIP-2 (37.5% and 15.9%, respectively). High IgG4 levels, jaundice, and other organ involvement are considered the most relevant risk factors associated with AIP relapse [75].

Nowadays, the approaches to maintenance steroid therapy significantly differ across the World since Western researchers opposed the routine use of maintained GC in cases of AIP with initial remission; the Japanese guidelines instead, suggest the administration of a GC maintenance therapy for 3 years in all patients with AIP-1 to reduce the risk of relapse. Recently, Yoon SB et al. documented that the relapse rate in patients with AIP-1 decreases with an extended duration of GC therapy up to 36 months [76].

However, the adverse events associated with long-term glucocorticoid therapy warrant reconsideration of this option.

### 4.2. Steroid-Sparing Agents

To avoid long-term side effects of glucocorticoid therapy, conventional glucocorticoid-sparing agents, including azathioprine, 6-mercaptopurine, mycophenolate mofetil, cyclosporine A, tacrolimus, methotrexate, cyclophosphamide, may be considered [23]. Available data supporting the use of one agent over another are limited, but the use of azathioprine (2–2.5 mg/kg body weight) is generally recommended. A recent meta-analysis found that azathioprine (AZA) was used in 85% of relapse cases [77] with the next most common therapy being mycophenolate mofetil (MMF). In a retrospective study of AZA use in AIP, De Pretis et al. confirmed the efficacy and safety of azathioprine and also showed that it was administered more frequently in AIP-1 patients Although there are no clinical trials on the use of AZA in AIP, its efficacy was investigated by Masaki et al. in a systematic review and meta-analysis; they found no AZA-treated patients naïve to corticosteroid treatment, but suggested the efficacy of AZA as maintenance therapy in patients with AIP who experience repeated relapses or are resistant to steroids. The meta-analysis had some limitations: All data collected were from Western countries, the dose used was different in each study (i.e., 50 mg/die or 100 mg/die), and it was not possible to classify AIP patients accurately (AIP-1 versus AIP-2) [78]. The efficacy of AZA and other immunomodulators depends on their ability to nonspecifically decrease B and T cell proliferation, which may be less effective than a therapeutic approach targeting Th2 cell response or regulatory T cell (Treg) activation [79]. It also examined which side effects occurred after AZA treatment; among them, pancreatitis, gastrointestinal symptoms, liver damage, severe leukopenia, and hair loss were the most common. The risk of acute pancreatitis after AZA treatment is relatively low, but the frequency of AZA-induced pancreatitis in AIP patients is unknown [80]. AZA-induced pancreatitis is a dose-independent adverse event affecting 2–7% of IBD patients treated with AZA; in a recent retrospective cohort study, Wilson et al. found that the HLA-DQA1-HLA-DRB1 polymorphism is an important marker of AZA-induced pancreatitis risk; when evaluating the HLA-DQA1*02:01-HLA-DRB1*07:01 polymorphisms, three groups can be distinguished:

(1) The extensive metabolizers with wild-type genotype
(2) The intermediate metabolizers with heterozygous variant genotype
(3) The poor metabolizers with homozygous variant genotype, who should therefore receive alternative treatment [81].

### 4.3. Rituximab

Rituximab (RTX) is a monoclonal antibody directed against the B-cell-specific antigen CD20 that acts as an efficient depleter of B cells in peripheral blood [82]. According to European guidelines, RTX should be considered in patients with AIP-1 if they are resistant to or cannot tolerate high-dose GC or have not responded to immunomodulatory therapies (IM). In patients with definite AIP-1, this monoclonal antibody could be considered as an alternative GC drug for induction therapy. It seems to be a promising steroid-sparing therapy, although long-term data are lacking [23]. Nikolic et al. analyzed the outcomes of RTX treatment in patients with AIP-1 in a study that included a large cohort of AIP patients. The indications for monoclonal antibody therapy were disease relapse, GC-refractory disease, IM or GC dependence, and, in one case, Castleman disease. Of the 103 patients with AIP, 11 received RTX and one received another monoclonal antibody, siltuximab (chimeric monoclonal antibody to IL-6), for Castleman disease. 66.7% of patients achieved complete remission and 33.3% achieved partial remission. None of them relapsed during the 17-month follow-up period. These results confirm that RTX is effective in the treatment of AIP-1 by inducing remission and preventing relapse [83]. In some special cases, rituximab can be continued as maintenance therapy. In one study, induction and maintenance therapy with rituximab was compared with induction therapy alone in IgG4-RD. Forty-five percent of patients treated with rituximab as induction therapy had relapsed, compared with only 11% of patients who also received rituximab as maintenance therapy. Relapse after rituximab therapy is more common in patients who are younger, have biliary disease (indicated by an increase in serum alkaline phosphatase), and have a higher IgG4 responder index score (an assessment tool that quantifies disease activity and response to treatment based on symptoms, laboratory and imaging findings, and urgency of treatment) [84]. These may be factors to consider when deciding whether or not to recommend maintenance therapy [85].

Rituximab has also been shown to be an excellent therapy for patients at high risk of relapse, such as those with multiorgan involvement, prior relapse, and failure of disease-modifying antirheumatic drug (DMARD) therapy. After two RTX doses (1000 mg each), 97% of patients achieved disease response that persisted at 6 months. This confirms that RTX therapy is highly effective in controlling disease without concomitant GCs [86]. In the study by Backhus et al., a higher number of relapses (61%) was observed in patients treated with rituximab as maintenance therapy, likely due to the long follow-up period of the study (71 months). In contrast to other studies, no predicted risk factors for relapse were found. We can therefore conclude that relapses remain common but can be managed with repeated RTX administration [87]. A high relapse rate was also reported in a French multicenter study confirming the efficacy of RTX in inducing remission and treating relapse in IgG4-RD but highlighting a high relapse rate after treatment (42%) [88]. An Italian study analyzed the average time of relapse in patients with AIP-1. In patients with relapsed AIP treated with two doses of rituximab (each administered 1000 mg 15 days apart and repeated after 6 months), 80% of them relapsed between the first and third year after starting therapy, with a median time to relapse of 30 months after the last infusion [89].

Compared with other traditional therapies, RTX is more efficient, even in patients who do not respond to previous immunosuppressive treatments. Soliman et al. documented that RTX has an efficacy rate of 94% in patients with recurrent AIP, whereas the efficacy rate of the other immunomodulators is significantly lower (67%) [90]. Similar results were provided by the Mayo Clinic study, in which patients with steroid intolerance or IM resistance treated with RTX achieved complete remission, with a success rate of 83.3%. Interestingly, in this study, infusion of rituximab was repeated every 2 to 3 months for 24 months (with a total of 8 additional doses) [79].

Backhus et al. reported that serum IgG4 levels decrease during clinical response to RTX treatment [87]. However, in approximately 20% of patients with AIP-1, serum IgG4 levels are normal at presentation, and IgG4 cannot be used to monitor therapy [91]. Monoclonal antibodies, such as RTX, can cause infusion-related reactions. Therefore, premedication with antihistamines, acetaminophen, and/or corticosteroids before each RTX dose is a common practice to prevent infusion reactions [86,87,92]. Two different dosing protocols have been described for treatment with RTX: 375 mg/m2 weekly for 4 weeks followed by maintenance infusions every 2–3 months (oncohematologic protocol) or two 1000 mg infusions 15 days apart every 6 months (immunologic/rheumatoid arthritis protocol) [23].

### 4.4. Other Biological Therapies

Although not among the therapies recommended in European and international guidelines, cases of patients with AIP treated with anti-tumor necrosis factor drugs (ANTI-TNF) have been described in the literature. In 2020, Lorenzo et al. described in a case report one of the first cases of AIP-2 autoimmune pancreatitis treated with adalimumab without the presence of inflammatory bowel disease. The patient, with a histologically confirmed diagnosis of AIP-2, who did not respond to rituximab treatment, received an induction sequence of three adalimumab doses without maintenance therapy. Steroids were discontinued, and the patient had no pancreatic recurrence after an 11-month follow-up period.

The same study also described three cases of patients with active IBD and recurrent AIP treated with anti-TNF therapy. Two patients were treated with adalimumab and one with infliximab. Pancreatic symptoms were controlled by induction treatment with anti-TNF therapy only, and no maintenance treatment was required. The characteristics of the patients are described in Table 5 [93]. This suggests that anti-TNF therapy may be a well-tolerated and effective second-line therapy for AIP-2 relapses or steroid dependence.

Another interesting case report suggests the efficacy of anti-TNF therapy in patients with AIP-I and IgG4 colitis as a specific extra-intestinal manifestation of the disease. The patient was a young woman with obstructive jaundice due to a pancreatic mass in which a biopsy revealed a benign inflammatory infiltrate. This condition was successfully treated with oral prednisolone, with complete regression of the mass. Subsequently, the patient developed colitis with high serum IgG4 levels and an IgG4-positive plasma cell infiltrate in her colon biopsies. After an initial response to oral therapy with prednisolone, symptoms recurred when steroids were discontinued. The patient did not even respond to maintenance therapy with AZA and rescue therapy with IFX. Adalimumab therapy was started at an induction dose of 160/80 mg followed by 40 mg weekly and resulted in a dramatic and sustained response. Three months later, sigmoidoscopy showed complete healing of the mucosa with histologic resolution of the IgG4 infiltrate.

Therefore, the contribution of ANTI-TNF seems to be relevant in IgG4-related diseases and could be tried as an emergency treatment [94].

Therefore, new therapeutic targets can be developed in patients with AIP and IgG4-RD. Monoclonal antibodies such as anifrolumab (a monoclonal antibody antagonist of the type 1 interferon receptor (IFNAR) developed for the treatment of systemic lupus erythematosus (SLE) and lupus nephritis [95] or sifalimumab (immunoglobulin G1-κ monoclonal antibody that binds and neutralizes most IFN-α subtypes)) [96] may be effective in these patients.

Additional drugs such as etokimab, which have IL-33 as a molecular target, may be another therapeutic option for patients with AIP and IgG4-RD [97].

Other promising treatment options include targeted therapies against B-cell lineage plasmablasts and CD4+ SLAMF7+ cytotoxic T cells [98].

These include inebilizumab (anti-CD19 antibody) and XmAb5871 (anti-CD19 with Fcγ-RIIb antibody), ianalumab (inhibitor of the B-cell activating factor family), abatacept (CD80/86 inhibitor), simtuzumab (anti-LOXL2 antibody), or daratumumab (anti-CD38 antibody), which may target B cells or plasmablasts.

Instead, elotuzumab (anti-SLAMF7 antibody) may be a potential approach against cytotoxic T cells.

Unlike rituximab, which induces B cell ablation, the anti-CD19 antibody inebilizumab can reversibly inhibit B cells. Inebilizumab was first approved in 2020 for the treatment of neuromyelitis optica spectrum disorder (NMOSD). The drug is currently being studied in clinical trials for desensitization in kidney transplantation, myasthenia gravis, and IgG4-related disorders [99].

## 5. Discussion

Autoimmune pancreatitis (AIP) is a rare form of pancreatitis with autoimmune features whose diagnosis is often insidious. When AIP is suspected, it is essential to distinguish between the two forms of it (AIP-1 and AIP-2), well-differentiated by the international consensus diagnostic criteria (ICDC) [1].

Underlining differences between these two entities reveals how they have very little in common. AIP-1 is the most common form of the disease worldwide and it is a pancreatic manifestation of immunoglobulin G4-related disease (IgG4-RD) [13]. In contrast, AIP-2 is a pancreas-specific disease not associated with IgG4 and typically without the overt extra-pancreatic organ involvement seen in type 1 [100].

In both cases, the clinical presentation is non-specific and is characterized by overlapping features with more sinister pathologies, such as pancreatic cancer, including jaundice, weight loss, and abdominal pain in some cases; for this reason, major efforts are required to find more specific tools to make differential diagnosis possible even without biopsy [101].

A better understanding of AIP pathogenesis is desirable to find new therapeutic targets and ameliorate the management of the two subtypes. The abnormal immune response is the cornerstone in the pathogenesis of AIP.

Innate immunity and adaptive immunity are involved in AIP-1 development. Through the production of IFN-1 and Il-33 M2 macrophages CD 163+ and pDCs [34,42] stimulate lymphocyte differentiation in TH2 and Tregs [44] which are responsible for the production of fibrogenic interleukins and of the immunoglobulins subclass IgG4 switch [48,49]. However, the role of IgG4 in the development of the disease remains unclear [53,54] and we do not know yet if certain triggers, such as alterations in gut microbiota, can promote the development of AIP-1. Much less is known about AIP-2 pathogenesis. Analyzing immunologic features of AIP-2, the only cytokine strongly expressed is IL-8 [66]. IL-8 is a chemotactic and inflammatory cytokine which causes angiogenesis and the chemotaxis of neutrophils. Periductal neutrophilic infiltration may lead to GELs. Interestingly, IL-8 overexpression was also identified in the crypt epithelium of ulcerative colitis, suggesting that there is a possible cross-linking immunologic mechanism in AIP-2 and IBD development.

The cornerstone of AIP treatment is steroid therapy. High doses of methylprednisolone lead to remission in most cases of AIP. However, relapses are quite common, especially in subtype 1; for this reason, the duration of steroid treatment is still debated and alternative therapies are sought to avoid the side effects of steroids. A good understanding of the pathophysiology of the disease may help us to discover possible potential targets for future treatment. Immunomodulatory therapy, such as azathioprine, 6-mercaptopurine, or mycophenolate, can be used as a steroid-sparing therapy to maintain remission. Azathioprine appears to be an effective drug for preventing relapse of AIP [80], but B-cell depletion therapy with rituximab showed a strong immunologic basis and proved to be an effective strategy for both inducing and maintaining disease remission and treating relapse. Rituximab guarantees complete remission in up to 83% of patients and is also an effective drug for the treatment of relapse and in patients with corticosteroid intolerance [102,103]. Immunotherapy represents the new frontier in the treatment of both types of AIP. Not only rituximab but also anti-TNF antibodies can help treat steroid-resistant forms of AIP-1 and its relapses [94]. Anti-TNF-alfa appears to be effective in AIP-2 [93], although relapse is much less common after steroid treatment than AIP-1 [75]. The literature on AIP-2 treatment with new biologic therapies is limited to specific situations.

## 6. Conclusions

AIP is a complex nosological entity, from diagnosis to treatment. Understanding the pathogenesis can help us find valid biomarkers and effective treatment. Available therapies are effective in most cases, but in the era of immunotherapy and personalized medicine, greater efforts should be made to find drugs that are as effective as corticosteroids in initiating and maintaining remission or treating relapse, but which have a more specific mechanism of action. The future perspective should focus on the identification of new biomarkers that allow accurate diagnosis and better differentiation between the different types of AIP and provide targeted therapy with fewer side effects.

## Figures and Tables

**Table 1:** Diagnostic criteria of autoimmune pancreatitis proposed by the Japan Pancreas Society (2006).

Diffuse or segmental stenosis of the main pancreatic duct with irregular wall and diffuse or localized enlargement of the pancreas by imaging studies such as abdominal ultrasonography (US), computed tomography (CT), and magnetic resonance imaging (MRI). High serum levels of γ-globulin, IgG, or IgG4, or the presence of autoantibodies, such as antinuclear antibodies and rheumatoid factor. Marked interlobular fibrosis and marked infiltration of lymphocytes and plasma cells in the periductal area, occasionally with lymphoid follicles in the pancreas.
For diagnosis of autoimmune pancreatitis, criterion 1 must be present, together with criterion 2, and/or 3. However, it is necessary to exclude malignant diseases such as pancreatic or biliary cancers. IgG: Immunoglobulin G; IgG4: Immunoglobulin G4

**Table 2:** Level 1 and Level 2 Criteria for AIP 1 [1].

Criterion	Level 1	Level 2
Parenchimal imaging Ductal imaging	Typical: diffuse enlargement with delayed enhancement (sometimes associated with rim-like enhancement) Long (>1/3 length of the main pancreatic duct) or multiple strictures without marked upstream dilatation	Indeterminate: segmental/focal enlargement with delayed enhancement (it also includes atypical findings, which include low-density mass, pancreatic ductal dilatation, and distal atrophy) Segmental or focal narrowing without marked upstream dilatation (duct size <5 mm)
Serology	IgG4 > 2x upper limit of normal value	IgG4 1–2 x upper limit of normal value
Other organ involvement	a or b a. Histology of extrapancreatic organs. Any of three of the following: -marked lynphoplasmacytic infiltration with fibrosis and without granulocytic infiltration -storiform fibrosis -obliterative phlebitis -abundant (>10 cells/HPF) IgG4-positive cells b. Typical radiology evidence At least one of the following: -segmental/multiple proximal or proximal and distal bile duct stricture -retroperitoneal fibrosis	a or b a. Histology of extrapancreatic organ including endoscopic biopsies of bile duct with both of the following: -marked lymphoplasmacytic infiltration without granulocytic infiltration -abundant (>10 cells/HPF) IgG4 positive cells b. Physical or radiological evidence of at least one of the following: -symmetrically enlarged salivary/lachrymal glands -radiological evidence of renal involvement in association with AIP
Histology of the pancreas	At least 3 of the following: -periductal lymphoplasmacitic infiltrate without granulocytic infiltration -obliterative phlebitis -storiform fibrosis -abundant (>10 cells/HPF) IgG4 positive cells	Any 2 of the following: -periductal lymphoplasmacitic infiltrate without granulocytic infiltration -obliterative phlebitis -storiform fibrosis -abundant (>10 cells/HPF) IgG4 positive cells
Response to steroid Rapid (<2 weeks) radiologically demonstrable resolution or marked improvement in pancreatic/extrapancreatic manifestations		

HPF: high-power field; IgG4: Immunoglobulin G4.

**Table 3:** Level 1 and Level 2 Criteria for AIP 2 [1].

Criterion	Level 1	Level 2
Parenchimal imaging Ductal imaging	Typical: diffuse enlargement with delayed enhancement (sometimes associated with rim-like enhancement) Long (>1/3 length of the main pancreatic duct) or multiple strictures without marked upstream dilatation	Indeterminate: segmental/focal enlargement with delayed enhancement (it also includes atypical findings, which include low-density mass, pancreatic ductal dilatation, and distal atrophy) Segmental or focal narrowing without marked upstream dilatation (duct size <5 mm)
Other organ involvement	/	Clinically diagnosed inflammatory bowel disease
Histology of the pancreas	IDCP Both of the following: -Granulocytic infiltration of duct wall (GEL) with or without granulocytic acinar inflammation -Absent or scant (0–10 cells/HPF) IgG4 positive cells	IDCP Both of the following: -granulocytic and lymphoplasmacytic acinar infiltrate -Absent or scant (0–10 cells/HPF) IgG4 positive cells
Response to steroid Rapid (<2 weeks) radiologically demonstrable resolution or marked improvement in pancreatic/extrapancreatic manifestations		

Combining the various criteria, the diagnosis of AIP-1 and AIP-2 could be Definitive or Probable [1]. IDCP: idiopathic duct-centric pancreatitis; GEL: granulocyte epithelial lesion; IgG4: Immunoglobulin G4; HPF: high-power field.

**Table 4:** U-AIP Criteria.

Negative pancreas cancer workup
2. Disease characteristics
2.1 Histology: typical histological features (AIP-1; AIP-2)
2.2 Imaging and serology: typical imaging findings and elevation of serum IgG4 other autoantibodies
3. Response to corticosteroids

Unifying Autoimmune Pancreatitis Criteria. To diagnose AP, it is necessary to respect criteria 1 with either 2.1, 2.2, or 3. U-AIP: Unifying-Autoimmune-Pancreatitis-Criteria; AIP: autoimmune pancreatitis; IgG4: Immunoglobulin G4.

**Table 5:** Characteristics of patients treated with anti-TNF.

Author, Year, [Ref.]	Patient’s Features	Type AIP	IBD	Treatment at AIP Recurrence	Anti TNF Therapy
Lorenzo D et al., 2020 [93]	Woman, 28 years old	AIP type 2	Negative for IBD	Rituximab	Adalimumab
Lorenzo D et al., 2020 [93]	Woman, 16 years old	AIP type 2	Ulcerative colitis	/	Adalimumab
Lorenzo D et al., 2020 [93]	Woman, 38 years old	AIP type 2	Crohn disease	Azathioprine and then methotrexate	Infliximab
Lorenzo D et al., 2020 [93]	Woman, 18 years old	AIP type 2	Crohn disease	Azathioprine	Adalimumab
Naghibi M et al., 2013 [94]	Woman, 16 years old	AIP type 1	IgG4 positive colitis	Azathioprine and then infliximab	Adalimumab

AIP: autoimmune pancreatitis; IBD: Inflammatory Bowel Disease; Anti TNF: anti Anti-Tumor Necrosis Factor; IgG4: Immunoglobulin G4.
